# Hybrid RF/VLC Network Architecture for the Internet of Things

**DOI:** 10.3390/s20020478

**Published:** 2020-01-15

**Authors:** Francisco Delgado-Rajo, Alexis Melian-Segura, Victor Guerra, Rafael Perez-Jimenez, David Sanchez-Rodriguez

**Affiliations:** Instituto para el Desarrollo Tecnológico y la Innovación en Comunicaciones (IDeTIC), Universidad de Las Palmas de Gran Canaria (ULPGC), 35017 Las Palmas, Spain; alexismeliansegura@gmail.com (A.M.-S.); vguerra@idetic.eu (V.G.); rperez@idetic.eu (R.P.-J.); david.sanchez@ulpgc.es (D.S.-R.)

**Keywords:** Internet of Things, personal area networks, visible light communications, network architecture, LoRaWan

## Abstract

In recent years, there has been a remarkable advance in monitoring technologies in many environments, be they urban or rural. These technologies, included in the Internet of Things (IoT) domain, allow remote control and acquisition of data from sensors for their subsequence analysis. All these systems are based on the interaction between sensors and actuators. To achieve this goal, it is necessary to provide a very high level of connectivity between the devices, especially as far as wireless systems are concerned. In this sense, there is a great variety of standards in the market of communication networks oriented to this end. One of the biggest challenges today is to allow inter-operability between these different technologies in order to homogenize this field. In addition to this, it is intended to introduce new communication techniques that can provide certain additional advantages to those already existing. The main idea is the creation of a cellular network where radiofrequency and optical technologies coexist, and whose link with the rest of the world is through long-range and low-consumption wireless technologies. The center of each cell, that is the lighting system, can be powered using solar panels, as can the existing systems in the market. The objective is that these panels are capable of providing the necessary energy to the rest of the necessary systems.

## 1. Introduction

Nowadays there are many remote monitoring techniques based on technologies framed within the Internet of Things (IoT) ecosystem. Nevertheless, there are still areas such as rural, maritime, or even ports, without high-speed radio coverage (GPRS, WiFi, etcetera). Although the possibility of monitoring environmental parameters is interesting and necessary in these areas, connecting them to the rest of the network is not easy. The inclusion of novel low-speed long-range communication technologies would enable the connection of these areas with an Internet access point, allowing the data produced by these sensors to be consumed on the network at low cost. Other types of wireless sensor communication technologies such as ZigBee allow for the automatic implementation of a wireless network within an area, interconnecting nodes over moderate distances with reduced power consumption [1,2]. Moreover, in many of the aforementioned scenarios, there is a possibility of finding a solar-powered lighting point. Therefore, a cell can be established at those locations, providing communication capabilities within its coverage area, as well as the interconnection of its elements with Internet or with a central node. Under these circumstances, it would also be possible to remotely operate actuators such as irrigation control, light dimming, and color temperature.

The main objective of this work was to develop a network architecture to cover these types of models (rural, maritime, or port environments) using low-power and long-range devices at moderate transmission speeds [3,4]. The main addressed challenge is the coordination of a hybrid network with several involved technologies. The system must allow inter-operability between different communication systems or standards in a transparent way to the user and the rest of the network. In this case, LoRa, ZigBee, and visible light communication (VLC) are explored as candidates to develop the described system.

**Figure 1:**
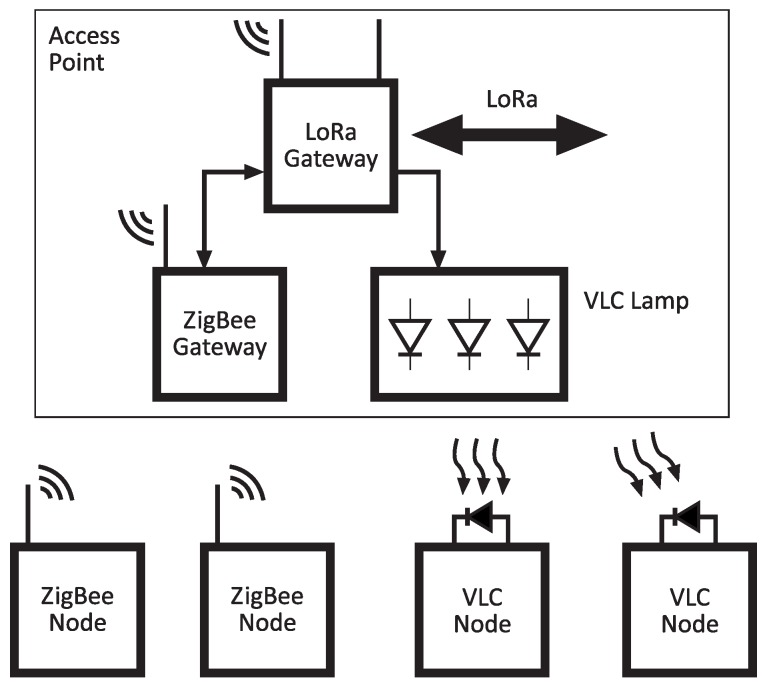
Cell communication between nodes using different technologies.

A cellular-type network structure has been developed. The backbone is performed by a low-power and long-range technology that uses long range (LoRa) modulation techniques. Figure 1 shows the cell network model. Each cell comprising the structure makes use of different technologies to provide node interconnection (both sensors and actuators). The considered technologies are low-range and energy-efficient:

- **ZigBee**. This technology is in charge of connecting the central node of each cell with the secondary nodes. It provides communications for actuators, as well as location information. Since ZigBee is low-power, the devices can be battery-powered and present a small form factor.
- **Visible Light Communications (VLC)**. VLC technology is based on the transmission of data by switching the lamp [5,6], in a way that is not visible to the human eye, and, in turn, allows two capabilities. One is the downlink communication between the lamp and the secondary nodes or between the lamp and an end user equipped with any mobile device (employing optical camera communications techniques) [7,8,9,10]. This technology also allows for the positioning of a mobile node [11,12,13] within its coverage. This latter capability could be especially useful in remote indoor environments such as greenhouses. Furthermore, a CMOS sensor could be added to implement the uplink of the VLC system.

In summary, the general objectives of this work can be broken down into the following:

1. offering low speed data coverage to remote areas for monitoring;
2. implementing a prototype that allows inter-operability of different technologies;
3. enabling location within the coverage areas of the cell;
4. allowing the network to transport and store digital contents (maps, images, audio, etc.) to be stored in the central nodes of each cell;
5. developing a star network topology that serves as a backbone to the cellular hybrid network.

The proposed hybrid network architecture implements a cellular network where both radiofrequency and optical wireless technologies are integrated into the IoT ecosystem. For that, three different models of communication under the low-consumption premise are used: Zigbee, LoRaWAN (employing the LoRa physical layer), and VLC. ZigBee and VLC networks are used to provide both sensors and actuators interconnection, and LoRa is used as the backbone of the system providing long-range communications.

The rest of this paper is organized as follows. Section 2 carries out a comprehensive state-of-the-art analysis in heterogeneous networks related to this work. Section 3 describes the proposed network architecture. In Section 4, the system design is explained, while the hardware implementation is described in Section 5. The experimental results are shown and discussed in Section 6. Finally, the conclusions are shown in Section 7.

## 2. Related Work

A considerable amount of works on heterogenous or hybrid networks for IoT can be found in the literature. In [14], Kirichek et al. proposed a heterogeneous architecture, but the interconnection between different technologies or the network backbone was subject to a pre-existing coverage area. In [15], Viacheslav et al. also proposed a heterogeneous IoT gateway computer appliance that provided interaction between different technologies and IP or non-IP networks. This work employed a cost-inefficient system, for example, using a computer to manage the HetNet; thus, it was not developed for low-power systems. Authors in [16,17] proposed named data networking (NDN) to allow node identification in an IoT architecture. NDN changes the semantics of a network service from delivering the packet to a given destination address for fetching data identified by a given name. Therefore, NDN provides a solution in which the devices no longer have a network address. However, it requires several additional elements, and its integration can be compromised when multiple networks are used. The system proposed in this work provides a unique frame format for all kind of networks to achieve both routing and identification, allowing a global routing method and an identification protocol, as is required in heterogeneous systems.

Jagannath et al. designed and implemented an end-to-end solution to enhance and enable public safety communication systems [18]. The system used heterogeneous wireless communication techniques (such as WiFi) to provide an access point for end-users, and LoRa to establish ad hoc networks covering larger areas. In addition, the authors proposed a cross-layer energy-efficient routing algorithm that aimed to maximize the network lifetime. Nevertheless, as was aforementioned, WiFi networks present serious constraints in terms of energy saving.

In [19], the authors proposed and evaluated an information monitoring approach based on NB-IoT and LoRa. The experiments were carried out in real scenarios, showing that the proposed system enables the achievement of wide-area information monitoring. Additionally, in [20], an LP-WAN-based architecture was proposed with the aim of monitoring the activities of Optimist Class sailboats and evaluating the performance of the LoRa protocol. Nevertheless, most works are not designed under the premises of low power, a low rate, and a long range, as the architecture proposed in this work. Both works were not capable of integrating other communication technologies of IoT, such as ZigBee and VLC.

Providing IoT connectivity in remote areas is still an open challenge. Krampe et al. designed and tested a hybrid network combining WiFi and Public Land Mobile Networks for providing internet access in precision farming applications [21]. The authors defined an ad hoc architecture to support the aforementioned RF technologies, and proposed a simple algorithm to decide opportunistically the communication interface. Nonetheless, the authors’ approach was mostly intended for monitoring applications.

The use of VLC (or optical wireless communication in general) combined with RF technologies has also been considered in the literature. For instance, authors in [22] implemented an asymmetric hybrid WiFi-VLC system in which the WiFi channel was only utilized as an uplink. This work showed that VLC downlink hotspots alleviate contention and interference on the RF channel. Furthermore, in [23], the authors proposed a hybrid WiFi-VLC system that comprised two links that can work simultaneously to provide aggregated bandwidth. One is a duplex WiFi link, while the other is an asymmetric duplex WiFi-VLC link where VLC is used as the downlink and WiFi as the uplink. It was shown that the approach was a good solution in terms of throughput. Nonetheless, in both works, the energy consumption was not evaluated, and as is well-known, WiFi network interface controllers impose serious constraints in terms of power consumption. Nevertheless, most works are not designed under the premises of low power, a low rate, and a long range, as the architecture proposed in this work.

Authors in [24] proposed an optimization problem that minimized the total energy consumed by LED lamps when used for data transmission. However, the system did not provide long-range communications such as that in the present work, where LoRa is used as the backbone.

Finally, Demirkol et al. proposed in [25] an architecture very similar to the one presented in this work. LiFi was proposed as an enabling technology for IoT in indoor environments. Nonetheless, the system does not allow for integration with other networks, and in order to provide a solution, the authors simply stated that the integration can be performed at the IP layer by defining an adaptation layer between IPv6 and the LiFi link layer. However, this should comprise header compression, optimized neighbor discovery, and fragmentation, and the authors do not provide any solution in this regard.

The work presented in this paper proposes not only a theoretical layered architecture as Demirkol’s, but also an actual implementation (including the cited fragmentation mechanism), demonstrating the feasibility of this heterogeneous network approach for IoT.

## 3. Network Architecture

**Figure 2:**
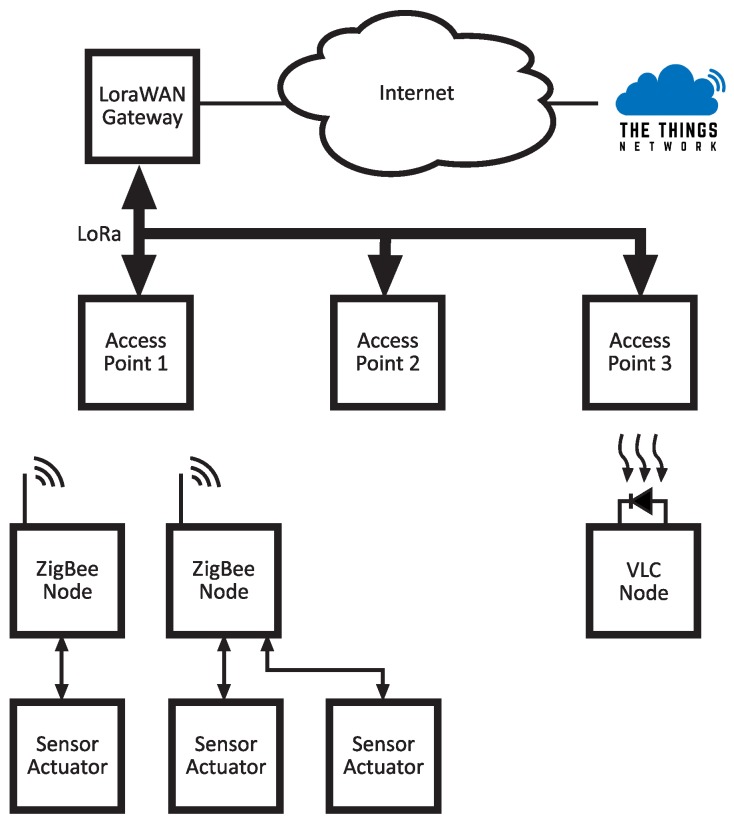
Global network architecture.

The proposed hybrid network architecture employs three different models of communication: Zigbee, LoRaWAN (employing LoRa physical layer), and VLC. For the implementation of these three communication methods, it is necessary to use different technologies for the inter-operability between the protocols used by each of them. Figure 2 shows the global network architecture implemented for the assembly of the developed hybrid network.

The first thing that can be observed in the network shown in Figure 2 is the set of sensor/actuator nodes. These nodes will collect the data of sensors that are connected and transmit these data to the access point, or receive data from it, to perform the response associated with the possible actuators connected.

In order to establish the interaction between nodes, in addition to communication between nodes and devices external to the sensor/actuator network, it is necessary to include a device that controls and establishes these communications. Therefore, to the network formed by the sensor/actuator nodes that use the Zigbee communication protocol, it is necessary to add a ZigBee coordinator device. Thus, it will be in charge of controlling the entire network and establishing not only communication between nodes, but communication between nodes and external devices, which will probably use another communication protocol.

The communication between cell nodes and the central network node is implemented using LoRa links, forming a star topology and allowing long distance communications between cells and the Internet network. One of the most important challenges of this work is the inter-operability between these two technologies. To achieve this, it is necessary to define a unique frame format which allows for node discovering, identification, and routing. This frame should also be compatible with the VLC link and should implement routing functions to redirect data or control messages between the different subnetworks. In this work, each technology is assumed as a subnetwork and each cell of the overall network. In order to achieve this, each of the access points implements a routing table with node identifiers and MAC directions.

The cell access point translates frames arrived from each technology and redirects them inside the cell to the IP network or to another cell.

One of the objectives of this work is to store digital content in the access point, which could be transmitted to a single user or to a node. For example, the central node could take a digital image and transmit it to the internet network using the LoRa backbone. To reach that objective, it is necessary to implement a segmentation system that takes into account the designed frame format, as will be seen later.

Packets received by the access point from the LoRa backbone are redirected to one of the two subnetworks (optical or ZigBee), transparently to the transport network and the user. Packets from these subnetworks are sent in the same manner.

With the inclusion of LoRaWAN in the coordinating modules of the sensor/actuator network, it is possible to create a LoRaWAN communication network. However, it is necessary to use a gateway forming a star topology at the backbone side, which, among other features, can establish communication between devices employing the same protocol, in addition to providing the user the possibility of uploading the data collected by the sensors to a cloud to facilitate its visualization, and allow for the possibility to interact with the actuators or devices located in the network.

In many cases, it is possible to use a previously available gateway at the area where one is working. However, there are areas that do not have this infrastructure. That is why in these areas it is necessary to implement a proprietary device. However, it is not only enough to have an Internet connection through the gateway with LoRaWAN, but a user interface and a service that stores the data collected by the network are necessary. To do this, an implementation is useful; thus, the user can not only visualize the data collected and sent through the LoRaWAN network, but can also interact with other networks and devices that are part of the subtotal of the hybrid network. This implementation is made with the service provided by “The Things Network” platform [26].

In addition, to create a more user-friendly environment, it is possible to add an integration with a service provided by some open platform such as TagoIO [27] so that the user can act with different devices, sensors, or actuators through the construction of dashboards, with which interaction is easier.

It should be noted that what is intended is that this architecture be as versatile as possible for the use of all types of sensors, locators, and control systems and is focused on remote environments without any type of coverage. Therefore, it is also useful to store data that can be updated in the central nodes of each cell, such as photographs, maps, and audio.

## 4. System Design

In this section, the designed architecture, the frame formats, and the segmentation and routing protocols are presented. In the first subsection, the developed protocol–abstraction layer and its frame format are described. This layer allows for the routing and identification of all nodes independently of their sub-network.

### 4.1. Protocol–Abstraction Layer

The data exchange used between devices of the same network is based on a defined frame format adapted for this communication protocol.

This is optimal and effective in the case of implementing a network with a single communication protocol. Nonetheless, in the implementation of hybrid networks with different involved protocols, some problems may appear when implementing the data gateway between endpoints. These problems are caused not only due to the different formats used in each protocol, but also due to the use of several parameters needed to properly perform communication, routing, and device identification in the hybrid network.

**Figure 3:**
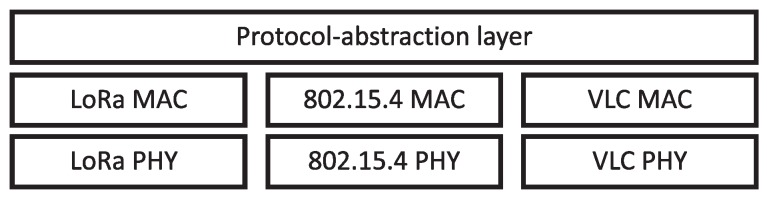
Diagram of the developed architecture.

For this reason, a solution based on the development of an abstraction layer that will define each of the aforementioned communication parameters in the hybrid network has been developed. The associated frame is defined in the same way regardless of the communication protocol. Thus, this frame will not only work for communication between ZigBee modules, e.g., but it will be used to communicate with any device on the hybrid network, without considering its sub-network or protocol. Figure 3 depicts a diagram associated with the protocol–abstraction layer developed in this work.

**Figure 4:**
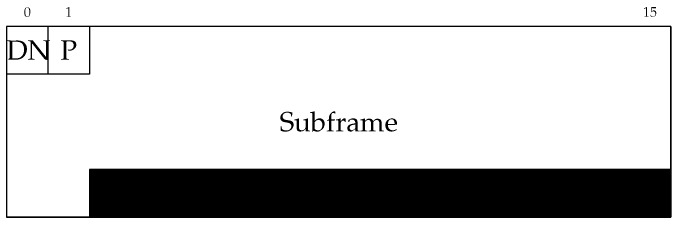
Hybrid network frame (HNF) structure.

The protocol–abstraction layer is supported by a technology-independent hybrid network frame (HNF) definition. The structure of this frame can be observed in Figure 4.

The DN field defines the packet’s destination network, which is hot-one encoded. In this work, only three bits have been used to indicate each one of the considered technologies (LoRaWAN (bit 0), VLC (bit 1), and ZigBee (bit 2)), and there are still 5 bits left that could be used to further enhance the capabilities of the developed architecture. In addition, part of the P byte (7 least significant bits) describes the frame’s purpose, which defines the payload interpretation (Table 1). The most significant bit of the P field indicates the presence of fragmentation. If this flag is active, it indicates that there are still data to be received. Nonetheless, if it is inactive, the frame transmission is over.

The encapsulation procedure of the HNF is the same regardless of the type of network or protocol used. However, it must be considered that not all the associated frames will be the same size in bytes. As an example, if a frame incoming from a ZigBee node were intended to be routed through a LoRaWAN link, this frame should be fragmented according to LoRaWAN’s maximum frame size. This size limitation has been integrated into the protocol–abstraction layer by establishing the subframe field’s maximum size to 48 bytes, with variable length.

### 4.2. Fragmentation and Routing

Data fragmentation is carried out using the same mechanism in the three technologies defined in the hybrid network architecture. It is based on the use of a flag defined by a bit of the HNF, as mentioned. When the data to be sent exceeds the maximum size established by the frame, it is fragmented and identified by activating the corresponding flag. This flag is set to a low logical level during the transmission of the last fragment of data, or if the amount of data to be sent does not exceed the mentioned maximum size.

**Figure 5:**
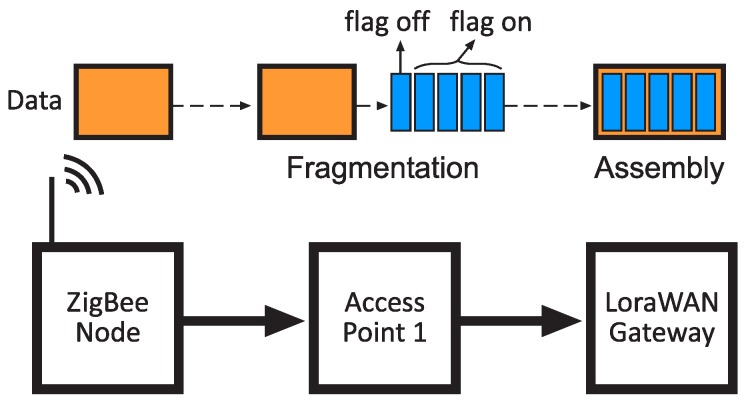
Fragmentation in a ZigBee-to-LoraWAN communication.

On the receiver side, the original frame is re-composed by concatenating all the incoming frames marked as fragmented, and finally adding the last one marked with the inactive flag. Figure 5 depicts the procedure in a ZigBee-to-LoRaWAN gateway communication.

Regarding routing, in this work, it was assumed that each node possesses knowledge about the whole network. Each node must know the MAC address of the coordinator module, and it must know the MAC addresses of each leaf node, which will be stored in an address table.

When inter-technology routing is needed (from a ZigBee node to a VLC endpoint), e.g., this action is performed by checking the HNF’s header (DN field). The access point decides the output gateway depending on the destination network, address, and purpose field. If the address corresponds to a node within the access point’s coverage, it performs internal routing. Otherwise, it relays the packet through its default gateway (LoraWAN in this work). The master coordinator (LoraWAN Gateway from Figure 2) of the system will decide, attending to the purpose field of the HNF, if the packet has to be sent to another access point or to a server on the Internet (sensor data). The opposite data path is also considered for interaction with actuators. In this case, a command arrives from the Internet and is properly delivered to the corresponding node.

### 4.3. ZigBee Integration

**Figure 6:**
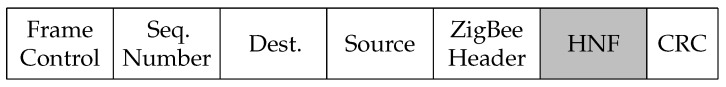
ZigBee packet structure with the HNF as payload.

The ZigBee protocol uses the packet structure defined in the IEEE 802.15.4 standard (Figure 6). This implementation of IEEE 802.15.4 defines both PHY and MAC layers, and ensures the transmission between two endpoints with known MAC addresses. In this work, there is an asymmetrical network knowledge, as has been already explained. However, a specific HNF purpose to enable communication between nodes within the same ZigBee network has been defined.

**Figure 7:**
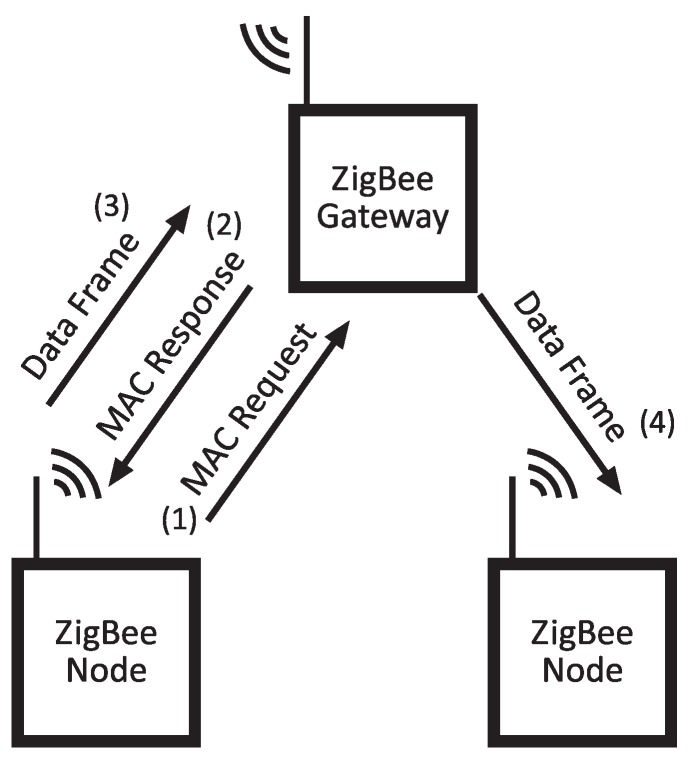
A diagram of an example of data exchange between same-network ZigBee nodes.

This purpose is the Data Frame (0x03), which concatenates the destination node ID and a variable length data field (1–47 bytes). Figure 7 graphs an example frame exchange diagram. In this figure, initial MAC request and MAC response packets have been included to illustrate the coordinator’s address discovery procedure.

In its current version, the system does not support ZigBee data exchange between nodes belonging to different subnetworks. However, this capacity could be easily introduced to modify the HNF frame by indicating not only the type of destination network but also a sub-network identifier (or a hybrid network address). Since the objective of this work is designing and implementing a multi-technology IoT application with a clearly defined backbone (LoRaWAN), this additional use case has not been considered.

### 4.4. VLC Integration

**Figure 8:**
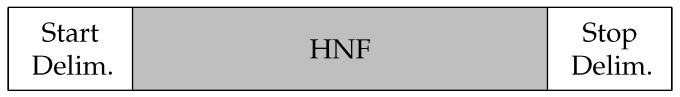
Visible light communication (VLC) packet structure with the HNF as payload.

IEEE 802.15.7m is VLC’s current standard [5]. This standard defines both PHY and MAC, but is not well suited for IoT since it defines a super-frame structure that assumes bidirectional optical links. In this work, VLC has been introduced as a downlink-only technology in broadcast mode, and a easy custom packet structure has been defined (Figure 8). The potential applications of unidirectional VLC links in IoT are well-studied due to the ubiquity of luminaires and lighting infrastructures. In this work, the communication with an indoor static sensor is demonstrated. Despite this, the proposed hybrid architecture is capable of providing VLC connectivity in other scenarios in which RF is not feasible (or is severely affected by a harsh environment). These scenarios comprise underground (mining, tunnels, e.g.) [28,29] and underwater environments [30], hospitals [31], and industry. Another interesting feature derived from the integration of VLC within the proposed architecture is the inherent positioning capability of this technology, which has been extensively studied in the literature [32].

Start and Stop delimiters have been introduced as a synchronization mechanism. This has been carried out because VLC systems have a dual use. On the one side, VLC access points must act as lighting devices, and this function must remain unaffected by data transmission. On the other hand, these devices are also data sources but information is not being sent continuously. The delimiters help in the time synchronization task. Furthermore, in this case, the HNF subframe (associated to the 0x05 purpose field) comprises only a variable-length data field (up to 48 bytes).

### 4.5. Sensor and Actuator Integration

**Figure 9:**
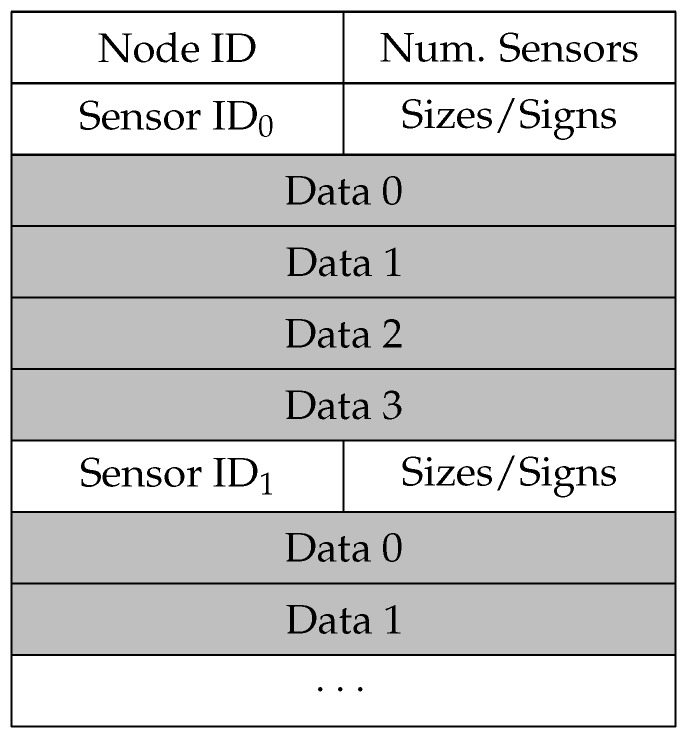
Board Sensor data and External Sensor data subframe structure.

The main objective of this architecture design is providing a supportive multi-technology hybrid network to communicate with sensors and actuators. This has been achieved by defining three different HNF purposes. 0x01 and 0x02 purposes correspond to Board Sensor data and External Sensor data transmission, respectively. The associated subframes have the same packet structure (Figure 9), but this division has been included to advance future necessities.

Node ID indicates the identifier of the data producer, which in this work corresponds to the ID stored by the ZigBee network coordinator. Num Sensors describe how many different sensor data are included in the frame. Sensor ID is the node’s internal identifier related to the sensor. The application layer will have the task of interpreting the type of sensor associated to this ID. The following field comprises 4 bits to indicate the sizes of each sensor data field, and 4 bits reflecting the signs of each one of them (positive: 0; negative: 1). Hence, the maximum number of sensor data allowed is 4. However, if fewer sensor data are being transmitted, the corresponding data fields set to 0x00 and the sign bit to 1 will show that there is no information.

**Figure 10:**
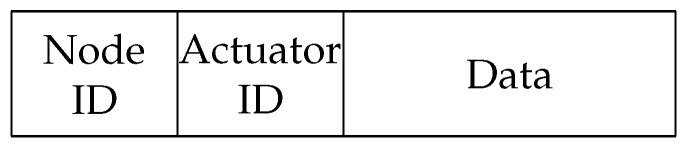
Interaction with the actuator subframe structure.

Besides sensor data packets, the 0x04 purpose indicates interaction with actuators. This frame definition states Node ID, Actuator ID, and Data (Figure 10).

## 5. Implementation

In this section, the physical implementation of the system is presented. The structure of each access point comprising the network is described first, followed by the global structure of the backbone, and finally the IP gateway.

### 5.1. Access Point Implementation

As has already been mentioned, this work was focused on implementing a hybrid network supporting VLC, LoRa, and ZigBee technologies. Both ZigBee and LoRa subsystems were constructed based on components-off-the-shelf (COTS) devices, while the VLC interface of the access point was custom-designed.

Libelium Waspmote kits were selected as main platforms because they contain a microcontroller, integrated sensors, secure digital memory (to store digital contents), and external ZigBee communication modules. The ZigBee module installed on the access point acted as coordinator of a ZigBee subnetwork, and in this work it had a hard-coded MAC address table. The ZigBee modules plugged into the sensor/actuator nodes had to be configured accordingly.

Low power consumption is a capital aspect that IoT networks must take into account. The technologies selected for this work comply with this requirement, and the remote nodes can be easily powered by batteries or even by solar panels. In addition, remote nodes can be set to sleep mode (or even deep sleep mode) in order to further increase energy saving.

Besides the initial configuration of the ZigBee coordinator module, the LoRaWAN module connected to the ZigBee coordinator (Figure 1) also requires some configuration. In this case, over the air activation (OTAA) was enabled in the LoRaWAN nodes. This type of easy configuration is similar to BlueTooth pairing, and is an easy, secure (is made at a very short distance prior to deployment), and fast way to establish the network.

Regarding the protocol–abstraction layer implementation, it has been carried out into the waspmotes’ embedded microcontrollers, using the frame formats previously described.

### 5.2. VLC Subsystem

With respect to the VLC subsystem, it was defined as a downlink-only technology in which a user could receive data from the LED lamp using the appropriate optical receiver. A VLC-based uplink has not been considered since this technology is intended to work for both data transmission and illumination, and a visible light uplink could produce discomfort. Although in this work no uplink was defined, it could be implemented using any infra-red band or other optical technology such as optical camera communication, in which high power emissions are not needed.

**Figure 11:**
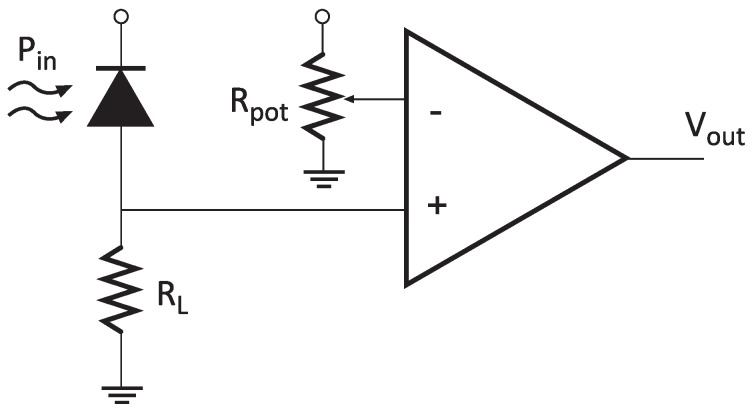
VLC receiver circuit diagram.

The implemented receiver was based on a positive-intrinsic-negative (PIN) photodiode and a high impedance amplifier followed by a comparator stage (Figure 11). It is important to note that the circuit presents a variable threshold level, which means it can be adjusted *ad hoc* depending on the background illumination level. The transmitter was based on the use of COTS devices. Both the LED lamp and the current driver were commercial, and the transmission was controlled by one of the waspmote’s digital pins and a MOSFET transistor. In order to avoid flickering, Manchester encoding was also implemented, resulting in a data rate of 1200 bps.

The signal-to-noise ratio (SNR) at the input of the comparator stage of Figure 11 can be observed in Equation (1).

(1) $$SNR=\frac{{\left(\int_{\lambda }P_{rx}\left(\lambda \right)R\left(\lambda \right)d\lambda \right)}^{2}}{2q\left(\int_{\lambda }P_{rx}\left(\lambda \right)R\left(\lambda \right)d\lambda +i_{d}+i_{b}\right)B+4k_{B}TBR_{L}^{-1}}$$

where $P_{rx}\left(\lambda \right)$ is the received optical power spectrum (Equation (2)), R(λ) is the photodiode’s responsivity, *q* is the electron charge, $i_{d}$ is the darkness current, $i_{b}$ is the background current, *B* is the bandwidth, $k_{B}$ is Boltzmann’s constant, *T* is the noise temperature, and $R_{L}$ is the high-impedance amplifier’s gain.

In VLC, the received power $P_{rx}$ depends not only on the line-of-sight (LOS) contribution but also on the non-LOS (NLOS) part of the optical channel impulse response. However, for LOS links, the NLOS contribution is usually residual and can be neglected. Regarding bandwidth, VLC supports hundreds of MHz in indoor applications [33]. Nonetheless, for IoT, these huge bandwidths are not needed, and coverage can prevail over throughput (which is purely limited by the frontends’ frequency responses).

(2) $$P_{rx}\left(\lambda \right)\approx \left\{\begin{array}{cc} P_{tx}\left(\theta ,\phi ,\lambda \right)\frac{A_{pd}\cos\left(\Psi \right)}{d^{2}}G_{lens} & \;\;\Psi \leq FOV/2\\ 0 & \;\;\Psi >FOV/2 \end{array}\right.$$

$P_{tx}\left(\theta ,\phi ,\lambda \right)$ is the emitter’s radiance depending on wavelength, ϕ is the azimuth, θ is the elevation, $A_{pd}$ is the receiver’s cross section, Ψ is the incidence angle, FOV is the receiver’s field of view, $G_{lens}$ is the optical gain, and *d* is the link’s range. In this work, no optical concentrator was used, and the FOV was limited by occlusion using a tube.

All the parameters of the implemented VLC subsystem are summarized in Table 2.

The operating base of the VLC transmitter circuit is the control of an LED lamp, through which the data are sent. The control of the LED lamp for communication through VLC is carried out for a common driver, which commutes the LED lamp, where the control pin corresponds to the output of data emitted by the VLC transmitter device.

**Figure 13:**
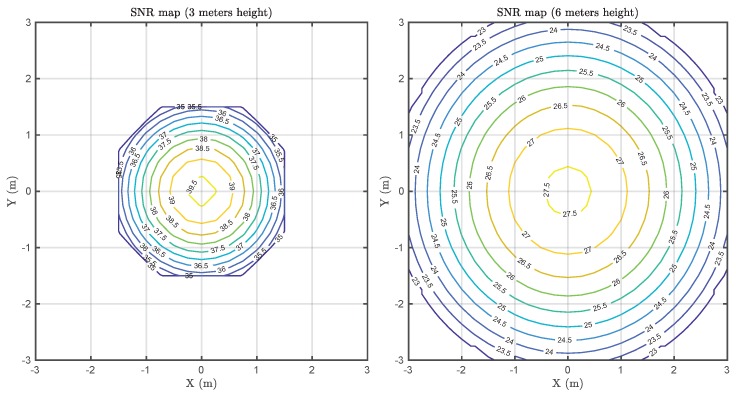
Signal-to-noise ratio (SNR) map at a 3 m height (**left**). SNR map at a 6 m height (**right**). In both cases, the receiver has been considered to point upwards.

VLC is a short-range technology capable of providing substantially high data rates. However, this technology is also interesting for IoT because of the ubiquity of LED lamps in both indoor and outdoor environments. Figure 13 depicts two estimated SNR maps at two different distances, taking into account the implementation parameters presented in Table 2. The left graph corresponds to a typical indoor environment, such as an office or a house, while the right graph could be considered representative of industrial environments. In both cases, the SNR within the coverage area (limited by the receiver’s FOV) leads to a BER metric below 10^−6^ (the worst case at the boundaries of the coverage area).

As mentioned above, VLC has been demonstrated to reach Gbps data rates [34]. Nonetheless, these results have generally been obtained under strict alignment [35] or laboratory conditions. Moreover, the used electronics is not cost-efficient, and the power consumption is not low-power compliant. In this work, since this architecture is IoT-oriented and is based on a LoRa backbone, the VLC data rate was selected to be close to the backbone’s speed in order to alleviate bottlenecking. In addition, this allows for a good SNR performance and robustness against misalignment.

### 5.3. Network Implementation

The next step is to establish communication between both Zigbee and LoRaWAN protocols. As mentioned above, the understanding procedure between these two protocols is based on the use of the defined HFN, since this will be the one used in both communication protocols.

**Figure 14:**
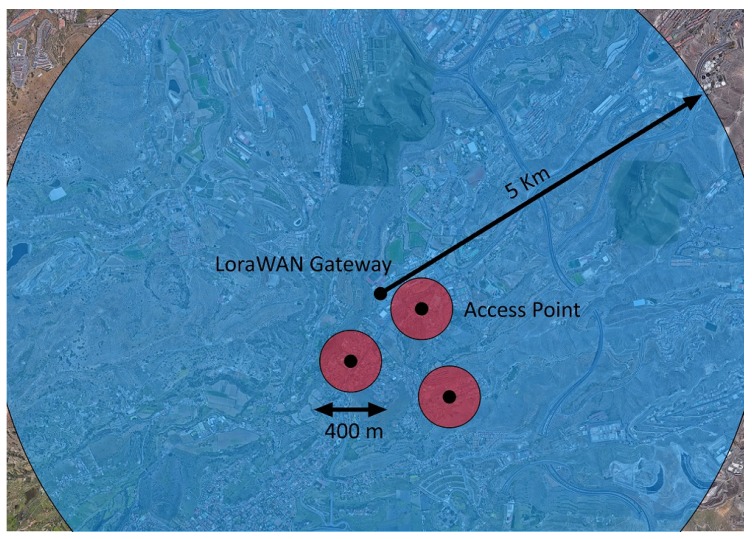
Simulated coverage maps approximated using the Okumura–Hata model for LoRaWAN (blue), and the general urban path loss (GUPL) model for ZigBee (red). The estimations are overlaid to the environment’s satellite image. The transmitter locations are tentative.

A coverage simulation using the Okumura–Hata model [36] for the LoRa protocol and the general urban path loss (GUPL) model for ZigBee [37] was carried out using the parameters of Table 3, where the experimental setup’s environment is assumed to be urban. The communication parameters were extracted from the nominal characteristics of the used components. The transmitter and receiver antenna heights are assumed to be similar to those used in an actual deployment (LoRaWAN gateway on top of a building and Access Points at street-light height). Figure 14 depicts the estimated coverage of the LoRaWAN gateway and several ZigBee coordinator nodes.

The LoRaWAN gateway has been defined to work through an implementation with The Things Network service and the TagoIO integration; thus, the user can view the frames and the associated data received in the LoRaWAN network, besides having the possibility of sending data destined to the devices to it.

In the VLC network, the data sent through the LED lamp is previously received through LoRaWAN. In order to achieve this, the corresponding access point has to send an empty frame to the gateway prior to sending the VLC frame. This must be performed in this way because it works with Class A modules, which, to receive data, needs to send a frame to open a reception window, allowing the LoRaWAN gateway to send the associated data.

Once the data is received, the HFN is extracted to subsequently carry out the communication via VLC with the LED lamp. Another possibility of this architecture is that the lamp can be used to download content that can be stored in the central node of the cell and updated through the LoRaWAN connection to the IP network.

Regarding the used LoRaWAN gateway, it has been configured in such a way that it establishes a link between LoRaWAN and the Internet, which allows for implementation with a user interface and a service that stores the data collected by the network. To do this, it is useful to add an implementation, so that the user can not only visualize the data collected and sent through the LoRaWAN network, but also can interact with the LoRaWAN network and the other networks and devices that are part of the sub-network. This implementation is made with the service provided by The Things Network.

### 5.4. Presentation Layer

In addition, to create a more user-friendly environment, it is possible to add an integration with a service such as that provided by TagoIO, so that the user can interact with different devices, sensors, or actuators through the construction of dashboards.

The part of the network formed by VLC comprises a transmitter module and a receiver module. For the correct operation of both endpoints, it is necessary to add external circuits to the modules used for VLC communication.

## 6. Results and Discussion

In order to verify the complete operation of the developed hybrid network, both a battery level and an accelerometer were used as sensor data inputs on the Waspmote development board with an information update period of two minutes.

The produced data was sent through the corresponding interface established in the LoRaWAN network, reaching its final destination (The Things Network) through the LoRaWAN–Internet gateway. Thanks to the implementation carried out with TagoIO, this information flux could be viewed using a user-friendly interface.

**Figure 15:**
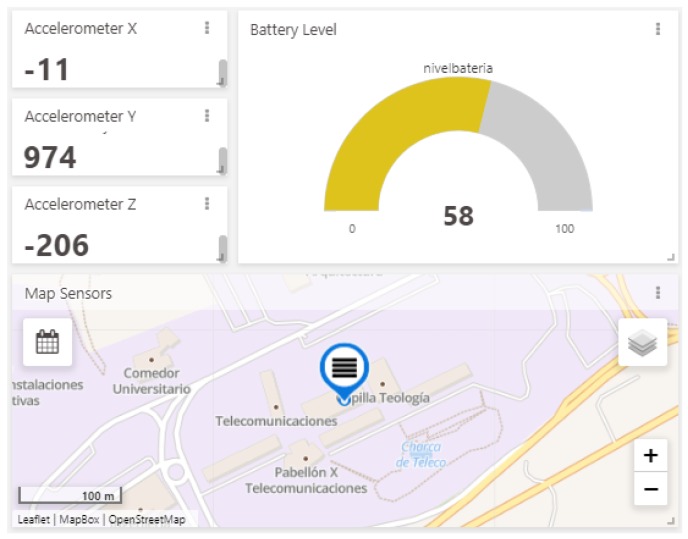
Sensors data received by LoRaWAN.

**Figure 16:**
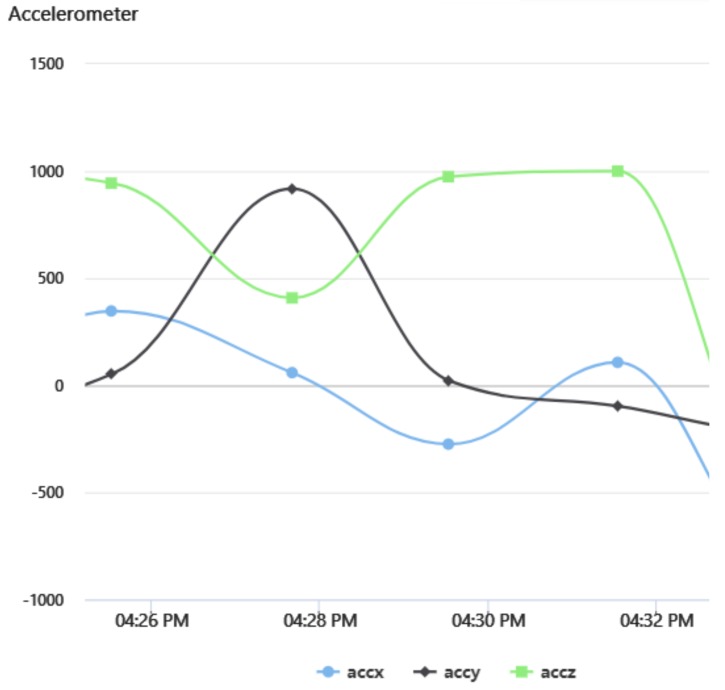
Accelerometer data changes received by LoRaWAN.

Figure 15 depicts the data on a dashboard built in the TagoIO service. Figure 16 shows the changes recorded by the accelerometer due to the movement of the sensor board, demonstrating both correct data acquisition and delivery by the hybrid network.

In order to assess the validity of the fragmentation procedure, a long-length phrase has been read from a text file, exceeding the maximum data size defined by the HNF, forcing the fragmentation of the information.

**Figure 17:**
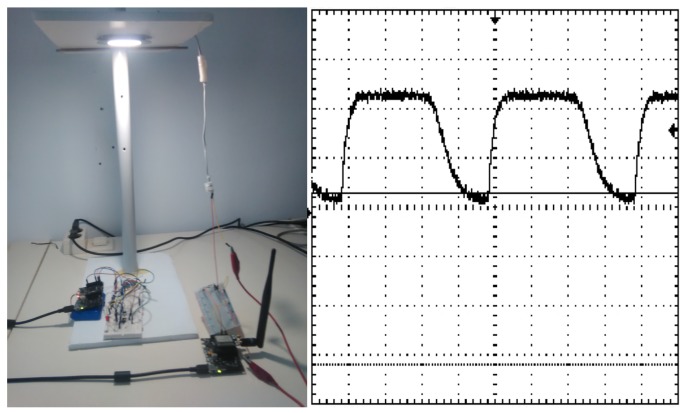
VLC data response through the hybrid network. Vertical resolution: 2 volts/div; horizontal resolution: 500 μs/div.

On the VLC communication side, as a first test, a small set of characters were sent from “The Things Network” platform with the objective of visualizing the appropriate changes in the receiver. An example of the signal recorded in the oscilloscope can be observed in Figure 17.

After assessing the proper functioning of the VLC interfaces, a more in-depth test was performed. One thousand VLC data frames (0x05 code in the purpose field of the HNF) with randomly generated characters were sent, as mentioned above. For simplicity, each HNF comprised a single character. No errors were detected on any packet within the coverage area of the testbed (Figure 17), suggesting a BER below 1.25 × 10^−4^.

Real-scenario experiments were performed on the complete system to validate error probabilities and propagation times. For verifying the correct functionality of the hybrid network, a series of tests were carried out. One of the performed tests consisted of sending several maximum-size packets between endpoints at different distances. In this test, segmentation was employed to send data without acknowledgment for the correct measurement of the packet loss rate.

**Figure 18:**
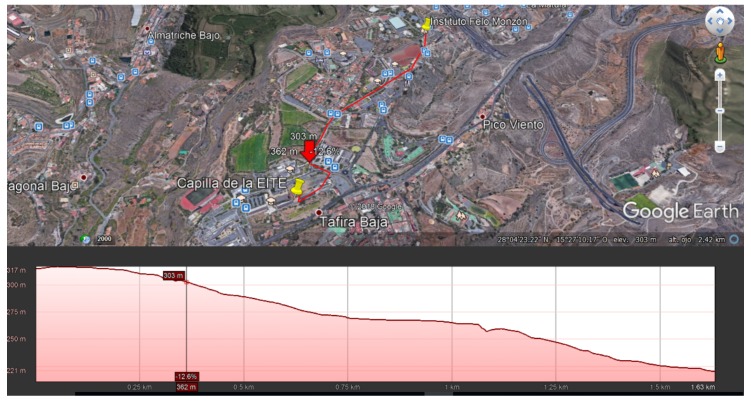
Approximate height profile of the test environment.

**Figure 19:**
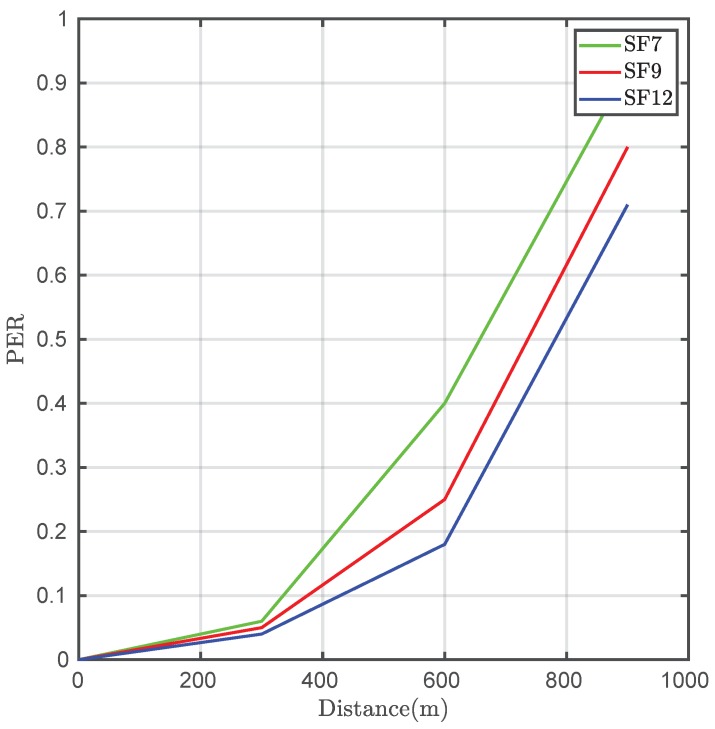
Error rate between sensor nodes and the gateway.

A total of 300 frames were sent at different distances in each test. The parameters employed for the LoRaWAN backbone were a bandwidth of 125 kHz and a spreading factor of 12. It is important to highlight that the tests were carried out in an urban area with a significant amount of buildings, and not in an open field without obstacles, which would have improved the obtained results (Figure 18). Moreover, the transmitter was located inside the laboratory, introducing an important indoor-to-outdoor additional loss. Figure 19 depicts the packet error rate (PER) versus the distance in this case. In an actual deployment in which the access point was anchored to a solar-powered LED luminaire, the SNR and hence the PER performance was considerably increased.

Another performed test was related to measuring transfer times. Concretely, the measured times corresponded to the data collection in the cloud-side of the architecture. Three hundred packets were sent through the complete network from the final nodes of the cell. Table 4 shows the average times for different distances and spreading factors.

As can be observed, the transmission time decreases in proportion to the diminishing of the spreading factor. However, there is an increase in the data error rate, being critical when the distance grows. Considering the obtained results and the purpose of the network, it is interesting to choose a high spreading factor value when coverage is capital, albeit the transfer time will be increased.

From the LoRaWAN central node, it is possible to send a packet to a node of the Zigbee network or a VLC node with a lamp for data transmission. With LoRaWAN Class A modules, at first it is necessary to send a frame to the LoRaWAN cell gateway, so that the module then opens a reception window to receive data. That is why we would have the same data rate and the same data flow in relation to time, although the reception of data through LoRaWAN and the subsequent sending to another network such as Zigbee or VLC is completed in less time, such as in the case of receiving data through LoRaWAN and sending it through VLC, assuming the use of a bandwidth of 125 kHz in LoRaWAN and a spreading factor of 12. To guarantee a long distance transmission and a frequency of 2 kHz in VLC communication, a delay of approximately 6 s was determined throughout the entire network.

Unlike other systems [38,39], in this case, inter-operability is based on the fragmentation capabilities offered by the HFN, not algorithms or payload encoding. This philosophy allows, in a simple way, the inclusion of other standards without substantially changing the infrastructure or the equipment layout.

## 7. Conclusions

In this work, as a major achievement, a hybrid cellular architecture has been developed that allows inter-operability between different technologies in the world of Internet of Things. The main application is to provide coverage to remote areas that lack mobile coverage or that require a long range and high data rates. The results obtained using the proposed network architecture suggest multiple applications such as long range communication, localization, monitoring, or even downloading content from a remote user. In order to address the aims of this work, a hybrid network architecture has been developed including the following aspects:

- A cell prototype has been implemented, and it includes a gateway between the ZigBee and LoRaWAN technologies that actsas a bridge in a transparent way to the user (human or machine) of the network. In addition, a central node that uses VLC technology capable of transmitting data on the downlink has been implemented in this cell. This technology also allows its use as a location mode in cases where other technologies such as GPS are not allowed.
- Moreover, a protocol–abstraction layer based on an HNF has been developed. The HNF allows routing, node identification, and discovery. Moreover, this frame format allows for segmentation of the transmitted data, so a download of larger content can be implemented between the IP network and each of the cells, and vice versa.

Furthermore, several field tests were carried out. These tests were based on demonstrating the feasibility and performance of the proposed network architecture. It was observed that the defined fragmentation scheme allows for long-length data transmissions regardless of the wireless technology. The proposed hybrid network architecture allows close-range technologies (ZigBee and VLC) to be used in isolated scenarios, such as rural environments, using the LoRa backbone in a transparent way.

## Figures and Tables

**Figure 12:**
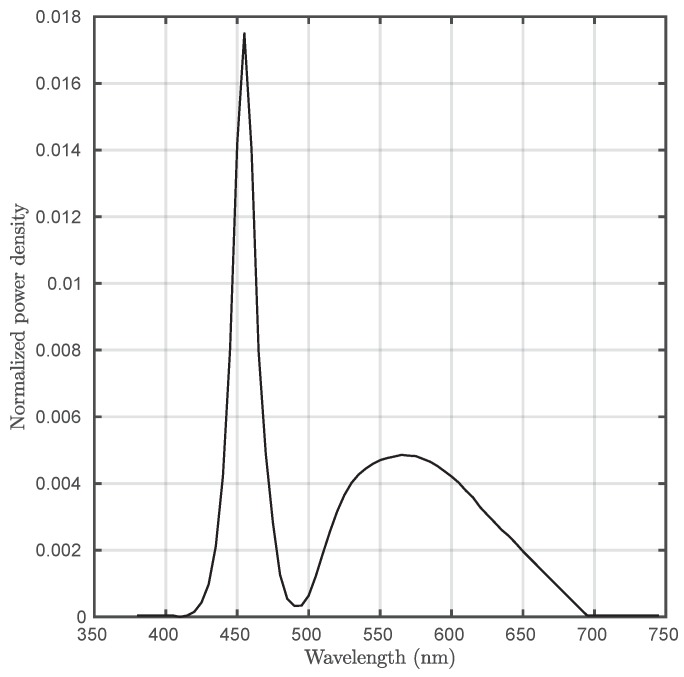
Energy-normalized optical spectrum of the used VLC lamp. This measure was obtained using an integrating sphere with a 30 cm diameter and an optical spectrum analyzer (BTS256 LED tester).

**Table 1:** Types of frames considered in this work.

Purpose	Description
0x01	Board sensor data (LoRaWAN)
0x02	External sensor data (LoRaWAN)
0x03	Data Frame (ZigBee)
0x04	Interaction with actuators
0x05	Data Frame (VLC)

**Table 2:** Parameters of the implemented VLC subsystem.

Parameter	Value
Tx Optical Power	≈1 W
Tx FWHM angle	60°
Tx spectrum	Figure 12
Rx FOV	60°
$G_{lens}$	1 (no concentrator)
$A_{pd}$	1 cm^2^ (Hamamatsu S1227-1010BR)
Noise Equivalent Power	6.7 × 10^−15^ W×Hz^−1/2^
$R_{L}$	1 kΩ
*B*	3 kHz
Data rate	1200 bps
Encoding	Manchester
Spectral efficiency	0.5

**Table 3:** Simulation parameters obtained from the developed demonstrator’s datasheets.

**LoRa**	
Tx power	14 dBm
Tx height	15 m
Rx height	5 m
Center frequency	860 MHz
Rx sensitivity	–134 dBm
Antenna pattern	5/4 Dipole
Antenna gain	4.5 dBi
**ZigBee**	
Tx power	0 dBm
Tx height	5 m
Rx height	1.5 m
Center frequency	2.4 GHz
Rx sensitivity	–100 dBm
Antenna pattern	5/4 Dipole
Antenna gain	5 dBi
**GUPL Parameters**	
Power component	2.2
Path loss exponent at 2.4 GHz	2.7
Attenuation constant at 2.4 GHz	0.06 dB/m

**Table 4:** Average time and error rate in the Zigbee–LoRaWAN link.

Spreading Factor	Range (m)	Average Time (s)	PER
SF12	300	6.2	0.03
SF12	600	6.4	0.19
SF12	900	6.7	0.72
SF9	300	5.4	0.03
SF9	600	5.5	0.26
SF9	900	5.7	0.81
SF7	300	4.6	0.04
SF7	600	4.7	0.4
SF7	900	4.9	0.92
